# Further Observations on the Liver Catalase Depressing Action of Tumours

**DOI:** 10.1038/bjc.1951.11

**Published:** 1951-03

**Authors:** D. H. Adams


### FURTHER OBSERVATIONS ON THE LIVER CATALASE DEPRESSING ACTION OF TUMOURS.

D. H. ADAMS.

*From the Cancer Research Department, London Hospital Medical College, London, E.1.*

Received for publication January 4, 1951.

RECENT work has provided evidence in favour of the supposition that tumours contain, and therefore probably release into circulation, a substance capable of depressing liver catalase activity. Nakahara and Fukuoka (1949) claimed to have produced a liver catalase depression in mice after the injection of alcohol-precipitated fractions obtained from human tumours. Similar fractions from normal tissue were stated to be inactive. Adams (1950*a*) commenced a study of the effect of the injection of homogenized mouse tumours into mice, and showed that after an early (24 and 48 hours) depression, the liver catalase level returned to normal approximately 4 days after injection, and fell once more as the new tumour grew. The results were interpreted as indicating that the initial depression is due to some substance or substances present in the injected material, the second depression only being due to the new tumour. Injection of homogenized normal tissues had no effect on catalase level.

Appleman, Skavinski and Stein (1950), in the course of an investigation of the variations in erythrocyte, kidney, and liver catalase activity in rats bearing the Jensen sarcoma or the U.C.L.A. fibrosarcoma, found that normal rats kept on a protein-free diet showed a drop in liver catalase activity to approximately 50 per cent of normal within 7 to 10 days from the time they were placed on the diet, and this low level was maintained for 53 days or longer. Two to three days after these animals had returned to a normal diet the liver catalase activity returned to normal. These authors consider their results in harmony with the alternative hypothesis, that tumours abstract from circulation some essential component required for the maintenance of a normal catalase level. However, the results already obtained in this laboratory, and those about to be presented, showing that a reduction of liver catalase level can readily be obtained in the absence of tumour growth, give definite evidence for the activity of some toxic material present in tumour tissue.

### MATERIALS AND METHODS.

### *Animals.*

The greater part of this work has been carried out using young adult mice (weighing 25 to 30 g.) of a stock albino strain. Two other strains have been used for individual experiments—FF fawn mice (Glaxo Laboratories) and albino mice obtained from the National Institute for Medical Research, Hampstead, known as the " Parkes " strain. During the period of the experiment the diet of the animals consisted of rat cubes and water *ad libitum*. As a routine the mice were starved for 12 hours before catalase determination.

*Tumours.*

(a) *Mouse.*—Sarcoma 37, Sarcoma 2146, Crocker sarcoma 180, Leukaemias FAK 1 and FAK 2, C57 sarcoma and Adenocarcinoma A, Strong A mammary carcinoma, CBA slow-growing sarcoma (kindly supplied by the Chester Beatty Research Institute, and the Imperial Cancer Research Fund Laboratories).

(b) *Rat.*—RIB 1 (slow-growing) and RIB 5 (fast-growing) sarcomata, originally produced in pure line albino rats by subcutaneous injection of a carcinogen, and kindly supplied by Dr. R. John (both tumours after *circa* 100 passages) ; Jensen rat sarcoma ; primary hepatoma produced by feeding aminoazobenzene, kindly provided by Mrs. C. Hoch-Ligeti.

(c) *Human.*—Oat-cell carcinoma of lung (kindly provided by Dr. Russel Brain) ; rectal carcinoma and breast carcinoma (kindly provided by Mr. D. V. Evans, F.R.C.S.). Fresh samples were taken from material removed at operation.

Tumour material was administered by the injection of a coarse suspension prepared with a TenBroeck grinder, except where otherwise stated.

*Liver catalase estimation.*

Catalase has been estimated by allowing liver homogenates to react with M/40 hydrogen peroxide in M/50 phosphate buffer (pH 6·8) at 0° C. as previously described (Adams, 1950*a*).

### RESULTS.

*Tumours which grow in the strains of mice used.*

The results already obtained had shown that in two strains of mice the injection of homogenized S37 tissue resulted in a marked depression in liver catalase activity 24 and 48 hours later, a rise to normal at about 4 days, and a further progressive fall down to about 30 per cent of normal with the growth of the new tumour (Adams, 1950*a*). This work has been repeated, using Sarcoma 2146 and the Crocker sarcoma 180. The variations in liver catalase level following the subcutaneous injection of coarse suspensions of these tumours into albino mice appear in Table I. Results in both males and females were similar to those previously obtained with S37 and Carcinoma 63 (Adams, 1950*a*), i.e., an early depression followed by a rise towards normal, and a subsequent fall with the growth of the new tumours. In no case was tumour growth observed before 5 to 6 days after injection.

No results are recorded after 7 days for the stock albinos injected with S2146, since tumours only appeared in approximately half the animals.

As previously observed with S37 and Carcinoma 63, the males appear more sensitive than the females. In fact, with S2146 and Parkes albinos there was little, if any, lowering of catalase level in the females. This differential effect has now been consistently observed with 4 tumours and 3 strains of mice.

*Effect of injection of disintegrated or partly centrifuged S37 sarcoma.*

It seemed of interest to attempt to produce a catalase depression in the complete absence of tumour growth. This would show beyond doubt that the depression must be due to some material contained in the original tumour tissue.

Two general methods have been employed: the first involved the injection of S37 sarcoma, which had been treated so as to render it incapable of giving rise to tumour growth, and the second, the results of which are presented in the next section, the injection of pure line mouse tumours which do not grow in the strains of mice used.

Sarcoma 37 tissue was disintegrated by shaking for several minutes with glass beads in a " Mickle " tissue disintegrator, made by Messrs. H. Mickle, Romeyn Road, Streatham, London. In this machine a specially shaped vessel vibrates with an amplitude of about 1 cm., 50 times per second. The material was then

Fig. 1.—Variation in liver catalase level after the subcutaneous injection of disintegrated Sarcoma 37 tissue into FF male mice (70 mg. per mouse). Points represent levels in individual mice, and the crosses the arithmetic mean values of the groups, in this and the following figures. Left-hand graph: Tissue disintegrated in normal saline. (Tumours appeared in 3/6 mice 12 days after injection.) Right-hand graph: Tissue disintegrated in M/100 citric acid. (No tumours grew.)
Ordinates: Catalase level in arbitrary units/mg. N.
Abscissa: Time in days.

injected into FF male mice. A stained film of the inoculum showed no intact cells. Fig. 1 is a plot of the subsequent variation in liver catalase level over a period of 10 days, the crosses representing the arithmetic mean values of the groups.

In the experiment represented in the left-hand graph the tissue was disintegrated in normal saline (pH 6·8), and it will be seen that a 24-and 48-hour depression was obtained, the level rising to normal at 4 days and remaining so at 7 and 10 days. However, this treatment was not completely successful in preventing tumour growth, in spite of failure to discover intact cells in the homogenate microscopically. Tumours appeared 12 days after injection in 3/6 mice set aside for observation. This was presumably due to the presence of a few intact cells in the injected material. The experiment was repeated, using M/100 citric acid as the suspending fluid (pH 5·5), and the results appear in the right-hand graph. The catalase depressing effect is very similar, but in this case no tumours appeared in 18 mice kept for 3 months, when the experiment was terminated.

A second method involved the partial centrifugation of a fine homogenate of S37 tissue in normal saline, prepared originally with a TenBroeck grinder. The suspension was centrifuged until approximately one-third of the suspended material (including all or nearly all the remaining intact cells) had sedimented. The cloudy supernatant was carefully removed, and injected into FF male mice, in amounts corresponding to approximately 70 mg. of original tissue per mouse. As shown in Fig. 2 a catalase depression was observed, which was somewhat smaller than that normally produced by injection of the same quantity of uncentrifuged material. Again, no tumours were observed in 6 mice kept for 3 months.

Fig. 2.—Variation in liver catalase level after the subcutaneous injection of partly centrifuged coarse homogenate of Sarcoma 37 tissue into stock albino male mice (dose equivalent to 70 mg. original tissue per mouse).
Ordinate : Catalase level in arbitrary units/mg. N.
Abscissa : Time in days.

*Effect of injection of tumours which do not grow in the strain of mice used.*

A number of pure line mouse tumours which do not grow, at any rate after the injection of coarse homogenates, in the stock albinos used, were injected subcutaneously or intraperitoneally in 100 mg. doses, and the liver catalase level determined at intervals. The results appear in Table II. In every case a significant depression in catalase activity was observed 48 hours subsequently, with a return to normal by the 4th to 7th day.

In one experiment of this series, not recorded in Table II, a different result was obtained. When C57 sarcoma was first used it must have become bacterially infected, since abscesses developed rapidly at the sites of injection. The catalase level at 48 hours was very low, and remained low at 4 days ; at 10 days, of the 6 remaining mice, 3 in which the abscesses were healed had high levels and 3

in which they were still discharging pus had low levels of catalase activity. In Fig. 3 the results of this experiment are compared with those of a subsequent one, in which non-infected C57 sarcoma was used. The depression produced by the infected material is much greater and more prolonged.

A number of rat and human tumours were homogenized and injected into mice. The results are given in Table II. No significant variations in catalase level were observed, and with the rat tumours in particular the variations appear to be completely random.

FIG. 3.—Variations in liver catalase level after the subcutaneous injection of coarse homogenate of C57 sarcoma into stock albino male mice (100 mg. per mouse). Left-hand graph : Accidentally infected tissue (causing abscess formation in under 24 hours). Right-hand graph : A subsequent experiment with non-infected tissue.
Ordinates : Catalase level in arbitrary units/mg. N.
Abscissa : Time in days.

In Fig. 4 catalase level (48 hours after intraperitoneal injection) is plotted against S37 tumour dosage. The points lie on a slightly curved line, showing a progressive decrease of catalase activity with dose.

The depression is seen to be approximately proportional to dosage over the range 10 to 30 per cent. Assuming that this holds for other tumours, a comparison may be made between the magnitude of the depressions given by those mouse tumours which grow, and those which do not, by correcting for variations dose.

The results of such a comparison appear in Table III, in which catalase depressions at 48 hours produced by different tumours are given, corrected to a 50-mg. in dose.

They indicate that the reduction in liver catalase activity produced by pure line mouse tumours which do not grow in the strain used lies between that produced by rat and human tumours (which had no effect) on the one hand, and by mouse tumours which do grow, on the other. The average depression of catalase 48 hours after injection given by the non-growing mouse tumours is approximately half of that given by the tumours which are capable of growth.

FIG. 4.—Liver catalase levels 48 hours after the intraperitoneal injection of varying quantities of coarse homogenate of Sarcoma 37 into stock albino male mice.
Ordinate : Catalase level in arbitrary units/mg. N.
Abscissa : Tumour dosage in mg.

TABLE III.—*Mouse Tumours. 48-hour Depression of Liver Catalase Calculated on Arithmetic Mean Values, and Corrected to a 50-mg. Dose.*

| Tumours which grow. | | Tumours which do not grow. | |
|---|---|---|---|
| Tumour. | Per cent depression at 48 hours. | Tumour. | Per cent depression at 48 hours. |
| S37 . . . | 23–35 | FAK I . . | 16 |
| M63 . . . | 24 | FAK II . . | 11 |
| S2146 . . . | 31 (stock albinos) | C57 sarcoma . | 10 |
| | 35 (Parkes albinos) | C57 adenocarcinoma A | 15 |
| | | CBA slow growing | 10 |
| | | Strong A mammary | 17 |
| Crocker sarcoma 180 | 27 | | |

### DISCUSSION.

The evidence so far presented in this and the previous paper (Adams, 1950*a*) in favour of the hypothesis that tumours contain a toxic substance capable of depressing liver catalase activity may be summarized as follows :

(1) Subcutaneous injection of coarse homogenates of 4 different tumours into male mice resulted in an early depression and rise to normal before signs of tumour growth appeared. Female mice showed a similar, though less pronounced, change.

(2) Subcutaneous injection of S37 tissue thoroughly disintegrated by shaking with glass beads in M/100 citric acid pH 5·5, and of a partially centrifuged S37 homogenate, resulted in depression of catalase activity, but no tumour growth.

(3) Subcutaneous or intraperitoneal injection of 6 pure line mouse tumours which do not grow in the stock albino strain used gave an early depression, followed by a rise to normal within 4 to 7 days.

It is not claimed, however, that nutritive effects play no part in producing the depression of catalase activity, particularly in animals bearing large tumours. Miller (1950) has recently shown that although catalase level in animals which are maintained on low or non-protein diets parallels approximately the level of total liver protein, the enzyme soon begins to disappear more rapidly than the remaining protein. Consequently, even if the only relevant effect of a large tumour on the nutritional status of an animal were a simple depletion of protein, a depression of catalase would result. However, the depression which occurs in the early stages of tumour growth may be expected to be due mainly to toxic material produced by the tumour, since the importance of purely nutritive factors, e.g., protein depletion, must be small at this stage, though becoming progressively greater as the tumour size increases. The effect of large tumours on catalase is probably the resultant of separate nutritive and toxic actions, which could not be readily distinguished.

The effect of injecting infected C57 sarcoma is quoted as a possible source of error in investigations of this nature. It is not yet known whether catalase depression is a common result of bacterial infections, but this possibility must be reckoned with when using, for example, human intestinal tumours, which are often infected. Greenstein (1947) states that infection of mice with certain strains of poliomyelitis virus, or with streptococci and staphylococci, has no effect on catalase activity. However, it has been observed in this laboratory during epidemics of a salmonella infection, and of ectromelia, that catalase level was low when the liver showed signs of involvement. Dounce and Shanewise (1950) have recently shown that catalase activity is significantly depressed in rats suffering from advanced murine leprosy. Apart from these isolated observations, no detailed investigation has been made of the effect on liver catalase of infections of the liver, or of injections of infected normal or malignant tissue.

Although a depression of liver catalase may occur in conditions other than cancer, this does not detract from the importance of the effect as a property of malignant but not of normal tissue, and work on the mechanism by which the toxic substance exerts its effect may throw light on some fundamental difference between normal and malignant tissue. Recently reports of preliminary experiments have appeared, indicating the importance of hormones in maintaining a normal level of liver catalase (Begg and Reynolds, 1950 ; Adams, 1950*b*). The present investigation is being followed up by an endeavour to relate the tumour-depressing effect with the hormonal control mechanism.

Depression of liver catalase activity has been shown to follow the injection into mice of mouse tumours, but not of rat and human tumours in single doses of up to 100 mg. Nakahara and Fukuoka (1949), on the other hand, observed a depression after the injection of alcohol-precipitated fractions of human tumours into mice. However, their dosage, referred to weight of original tissue, was probably very much higher than that used here. All that is now claimed is that injection, in the dose used, of mouse tumours into mice produces a considerable depression of liver catalase, while injection of similar doses of tumours of other species produces no apparent change. The precise significance of the observed specificity is not clear. The fact that mouse tumours which do not grow give effects intermediate between those of mouse tumours which grow and tumours of other species, suggests that the capacity of a tumour for growth in a particular host may be connected with its catalase depressing activity. Greenstein and

Ancervont (1942) have already stated that, in general, tumours which grow rapidly give larger catalase depressions than those which do not. Possibly the catalase depressing substance exercises some protective effect on the tumour itself. At any rate, since the catalase depressing substance is capable, in relatively small dosage, of affecting so greatly at least one liver enzyme, and probably others, its influence on the enzyme pattern in the tumour cells, where its concentration must be far higher than in the circulation, may well be profound.

### SUMMARY.

(1) Injection of coarse homogenates of Sarcoma 2146 and Crocker sarcoma 180 in 50-mg. doses gave significant depressions of catalase level 48 hours after injection followed by a rise to normal at 4 days, as had previously been observed with Sarcoma 37. No tumour growth was observed earlier than 6 days after injection. Males were more sensitive than females. The early variation in females was not significant in one case.

(2) Injection into stock albino mice of Sarcoma 37 tissue (*a*) disintegrated in M/100 citric acid, or (*b*) partly centrifuged after coarse homogenization in 70 mg. doses, resulted in a depression of liver catalase without any observable tumour growth.

(3) Six pure line mouse tumours, which do not grow in the stock mice used, also gave catalase depressions 48 hours after injection in 100-mg. doses, followed by a return to normal at 4 or 7 days.

(4) Rat and human tumcurs in 100-mg. doses had no effect on mouse liver catalase.

My thanks are due to Professor S. P. Bedson for his interest, and to Dr. M. H. Salaman for his suggestions and advice. I am also indebted to Mr. L. J. Hale for skilled technical assistance, and to Mr. G. Downes for his care of the animals. Acknowledgments for the supply of tumour material are given in the text. I would, however, like to thank Mr. J. Marsh, of the Chester Beatty Research Institute, and Miss Griffiths, of the Imperial Cancer Research Fund Laboratories, for their helpfulness in providing a number of mouse tumours.

The expenses of this research were partly defrayed out of a block grant from the British Empire Cancer Campaign.

### REFERENCES.

ADAMS, D. H.—(1950*a*) *Brit. J. Cancer*, **4**, 183.—(1950*b*) *Nature*, **166**, 952.

APPLEMAN, D., SKAVINSKI, E. R., AND STEIN, A. M.—(1950) *Cancer Res.*, **10**, 498.

BEGG, R. W., AND REYNOLDS, E. F.—(1950) *Science*, **111**, 721.

DOUNCE, A. L., AND SHANEWISE, R. P.—(1950) *Cancer Res.*, **10**, 103.

GREENSTEIN, J. P.—(1947) ‘Biochemistry of Cancer.’ New York (Academic Press).

*Idem* AND ANDERVONT, H. B.—(1942) *J. nat. Cancer Inst.*, **2**, 345.

MILLER, L. L.—(1950) *J. Biol. Chem.*, **186**, 253.

NAKAHARA, W., AND FUKUOKA, F.—(1949) *Gann*, **40**, 45.


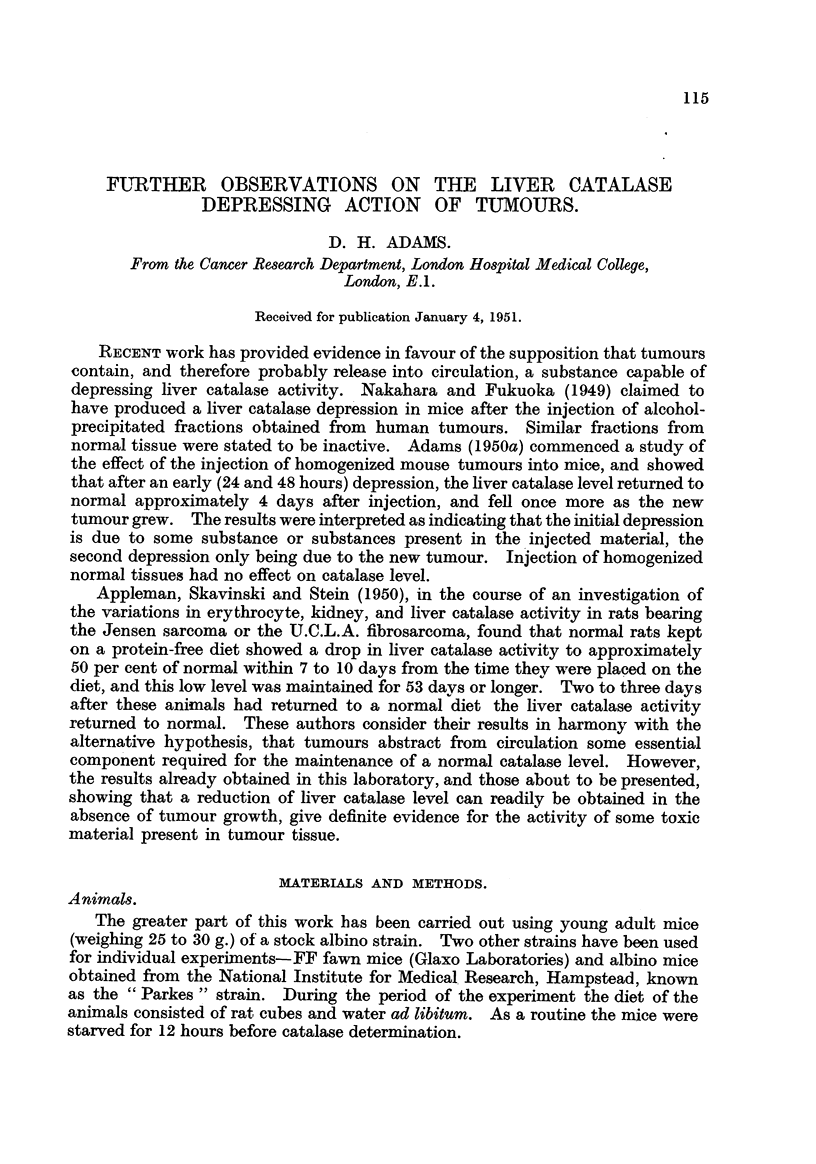

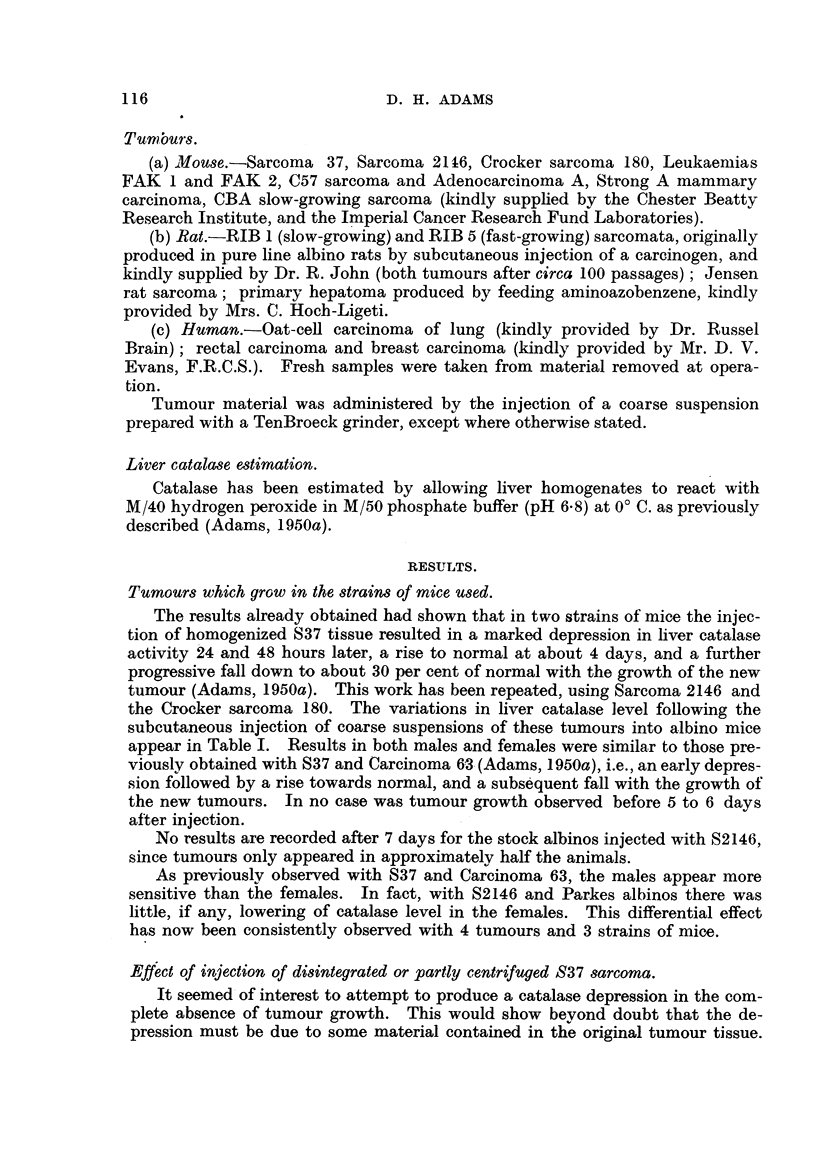

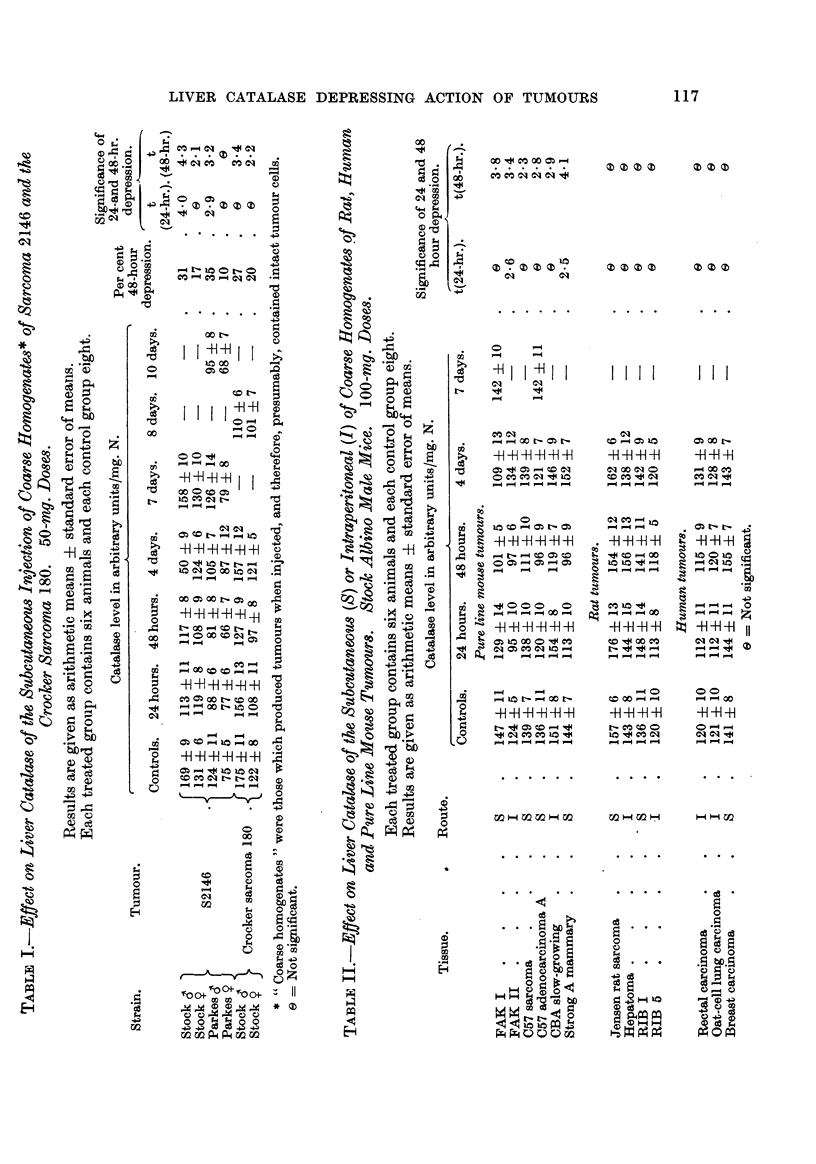

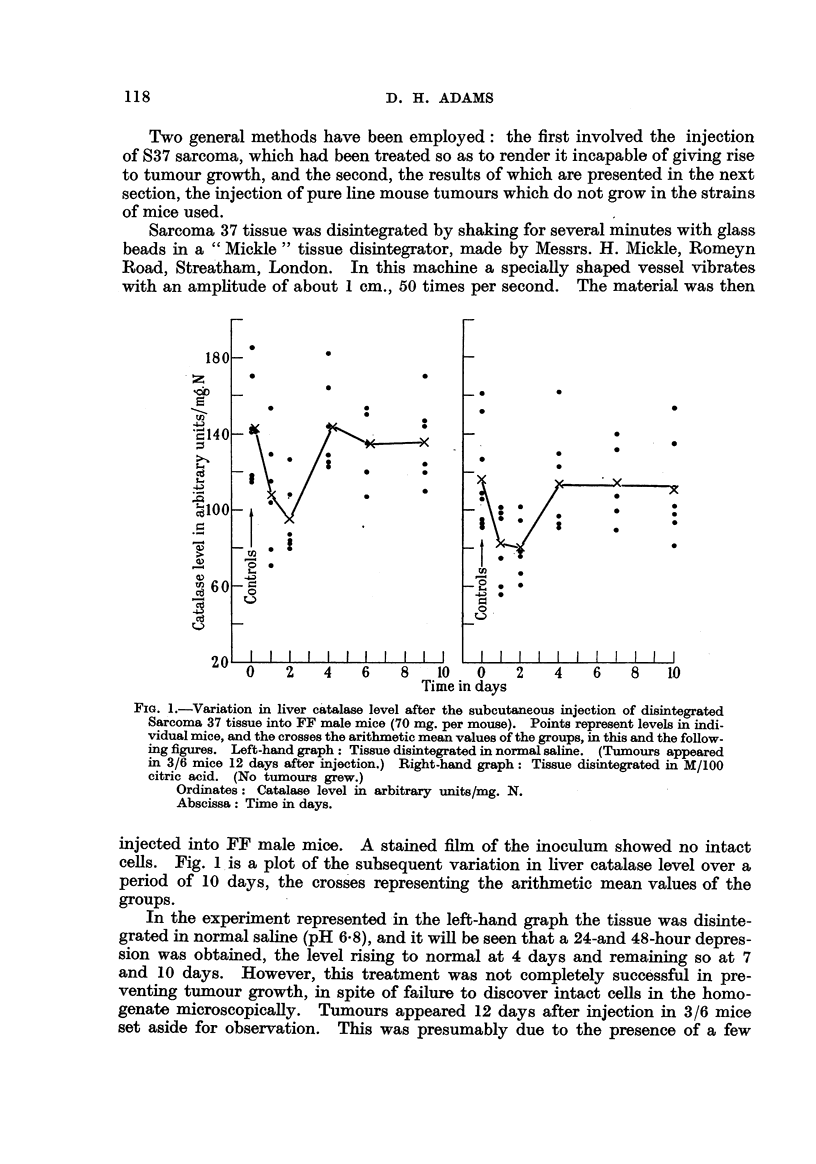

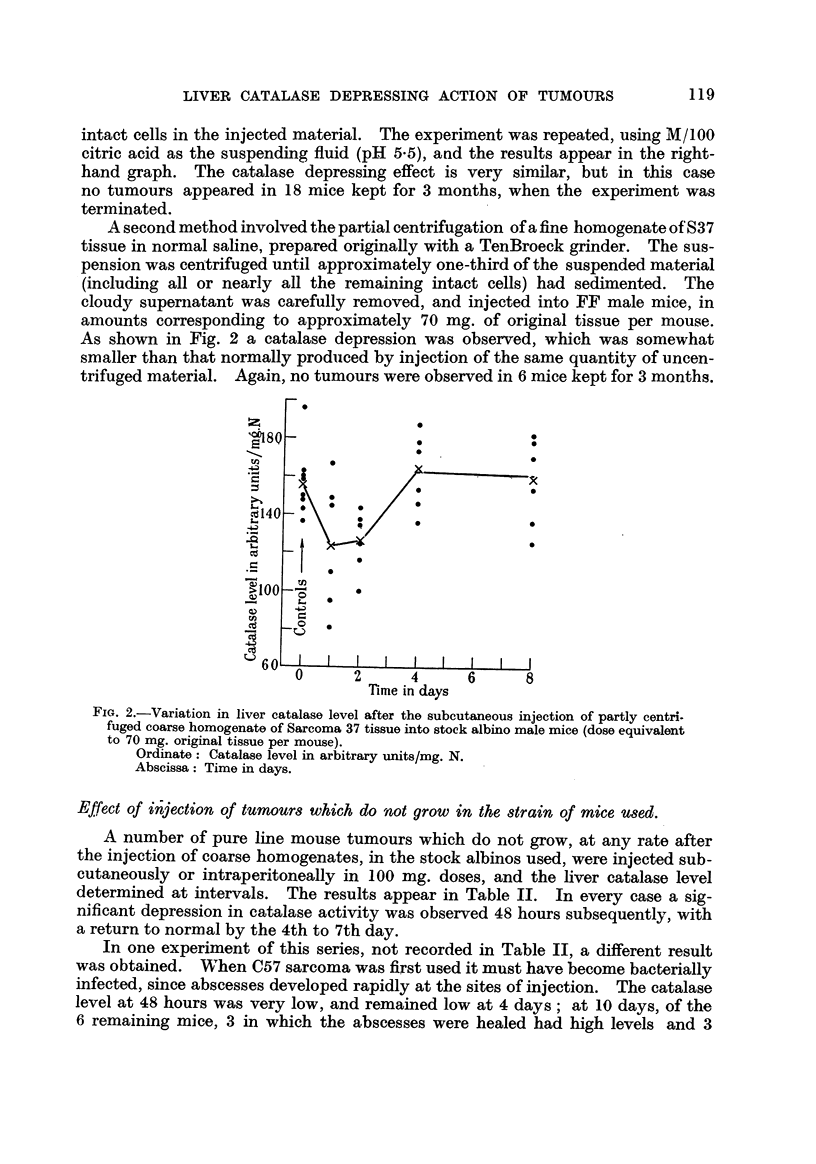

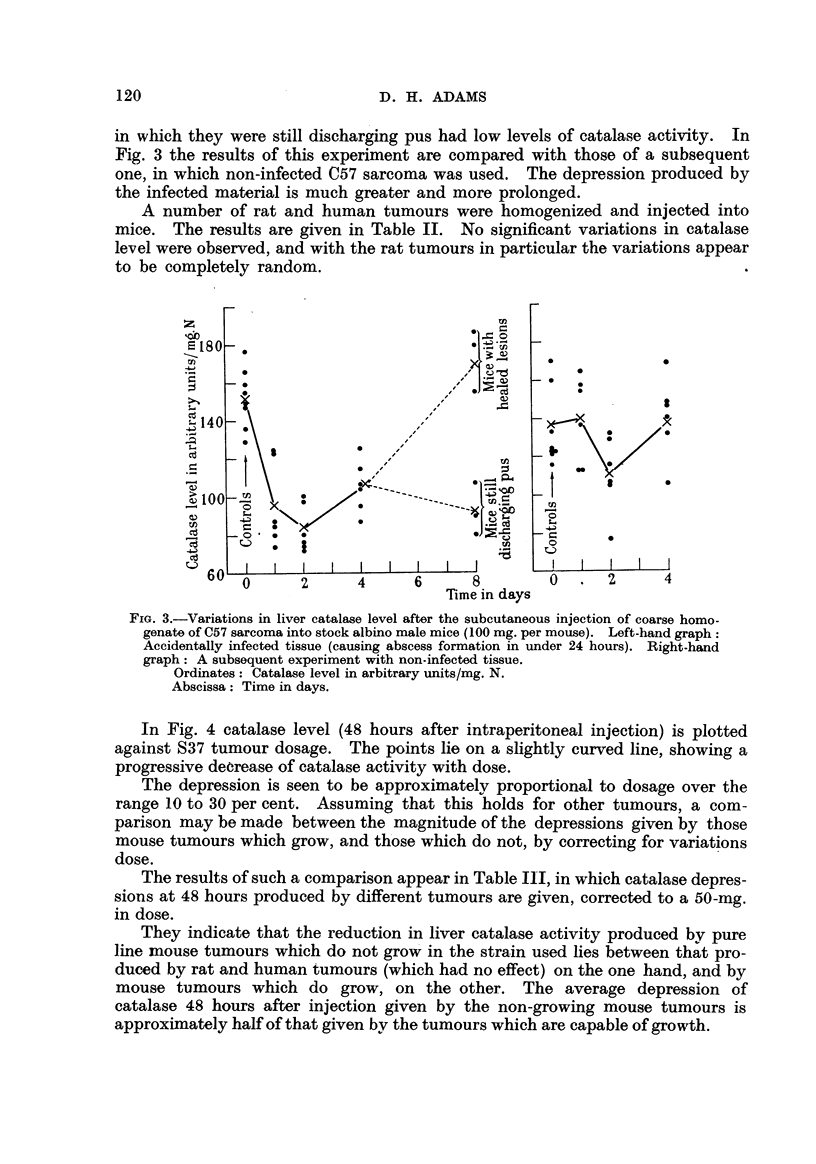

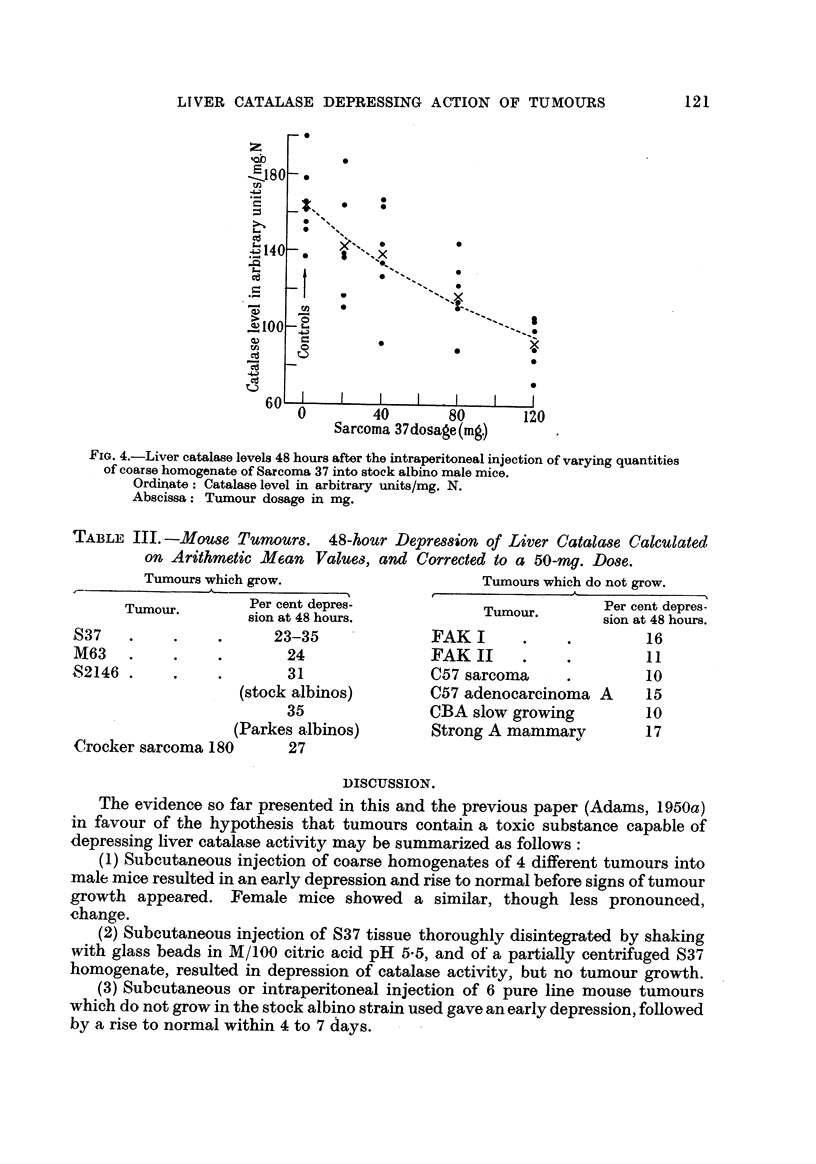

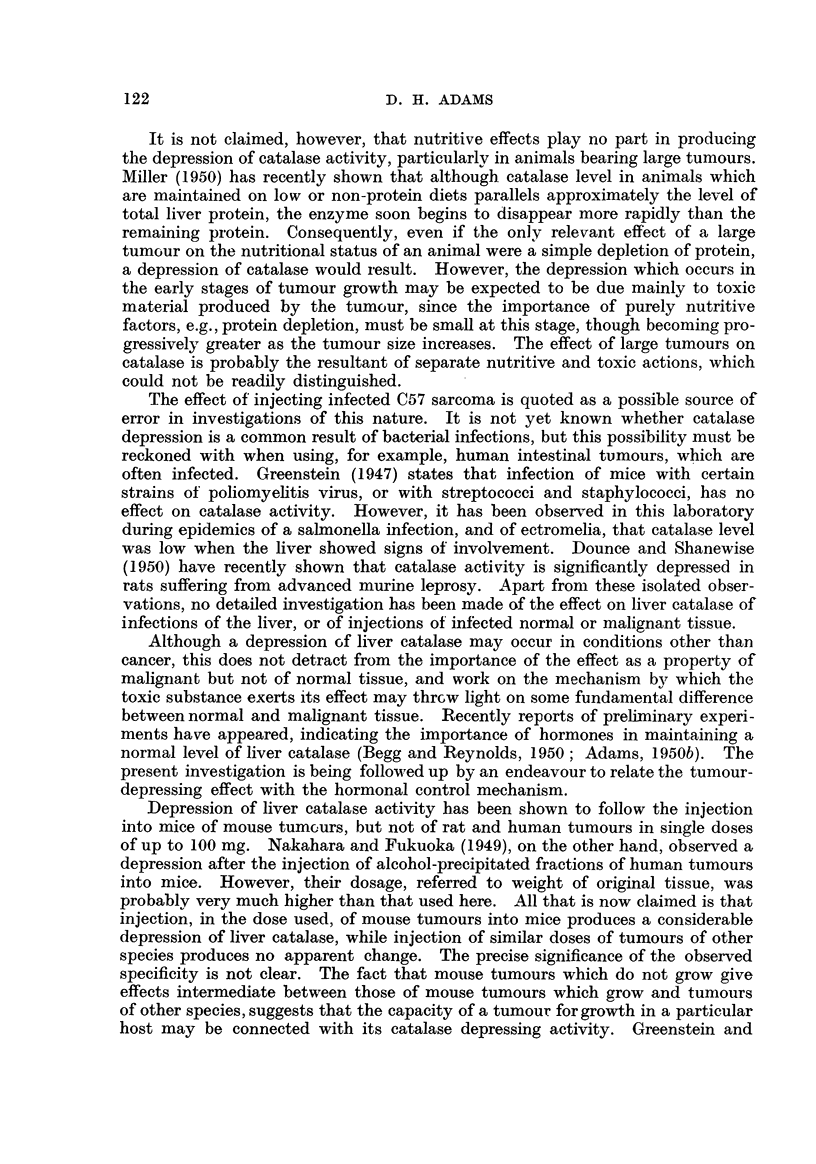

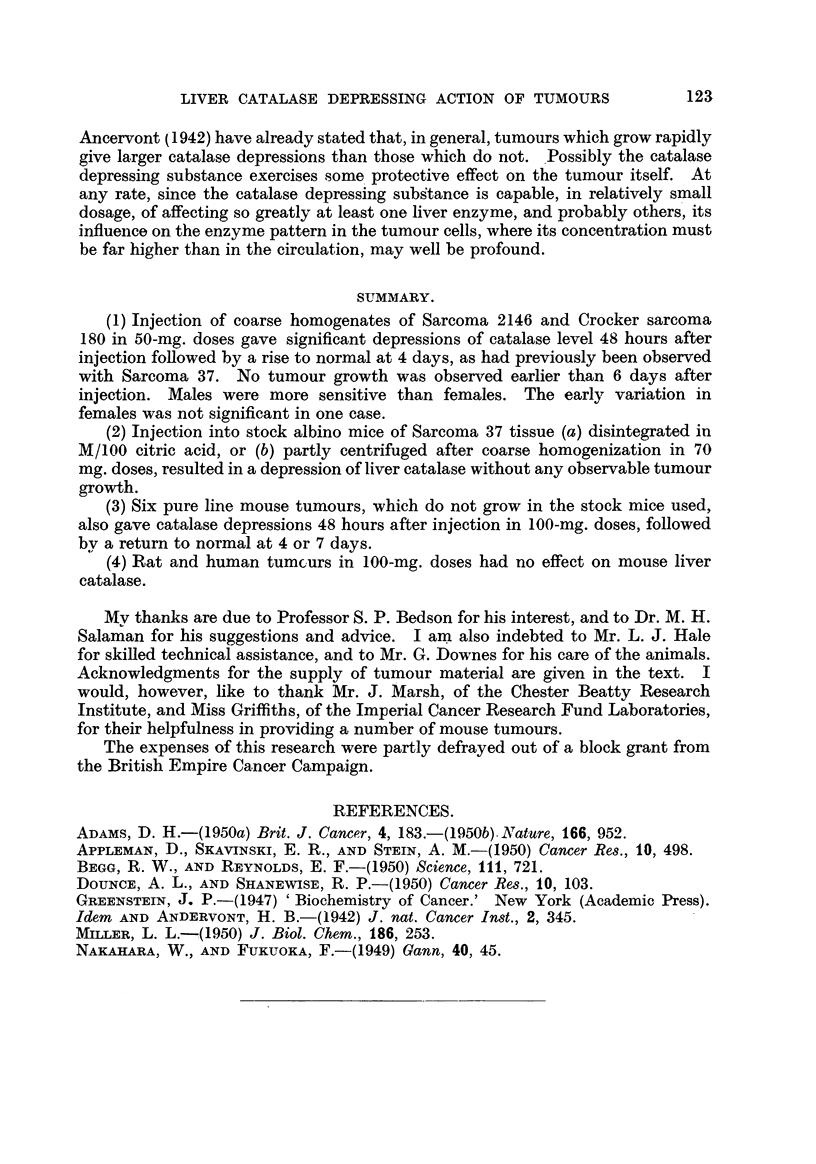


## References

[OCR_01255] APPLEMAN D., SKAVINSKI E. R., STEIN A. M. (1950). Catalase studies on normal and cancerous rats.. Cancer Res.

[OCR_01256] BEGG R. W., REYNOLDS E. F. (1950). Effect of adrenalectomy on liver catalase activity in the rat.. Science.

[OCR_01262] MILLER L. L. (1950). The loss and regeneration of rat liver enzymes related to diet protein.. J Biol Chem.

