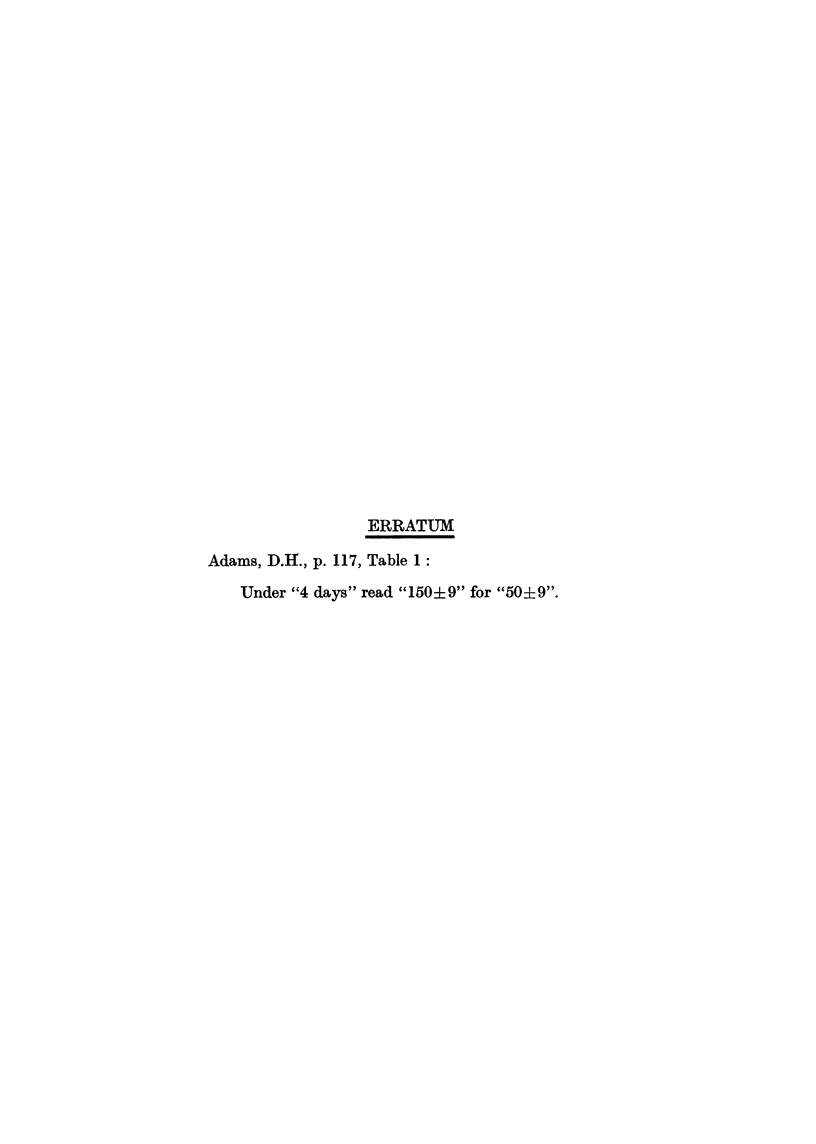# Erratum

**Published:** 1951-09

**Authors:** 


### ERRATUM

Adams, D.H., p. 117, Table 1 :

Under "4 days" read "150$\pm$9" for "50$\pm$9".